# Nativity-Related Disparities in Preeclampsia and Cardiovascular Disease Risk Among a Racially Diverse Cohort of US Women

**DOI:** 10.1001/jamanetworkopen.2021.39564

**Published:** 2021-12-20

**Authors:** Ellen Boakye, Yaa Adoma Kwapong, Olufunmilayo Obisesan, S. Michelle Ogunwole, Allison G. Hays, Khurram Nasir, Roger S. Blumenthal, Pamela S. Douglas, Michael J. Blaha, Xiumei Hong, Andreea A. Creanga, Xiaobin Wang, Garima Sharma

**Affiliations:** 1 Ciccarone Center for Prevention of Cardiovascular Diseases, Division of Cardiology, Johns Hopkins School of Medicine, Baltimore, Maryland; 2 Department of Medicine, MedStar Union Memorial Hospital, Baltimore, Maryland; 3 Division of General Internal Medicine, Johns Hopkins School of Medicine, Baltimore, Maryland; 4 DeBakey Heart & Vascular Center and Center for Outcomes Research, Houston Methodist Hospital, Houston, Texas; 5 Division of Cardiology, Department of Medicine, Duke University School of Medicine, Durham, North Carolina; 6 Department of Population, Family and Reproductive Health, Johns Hopkins Bloomberg School of Public Health, Baltimore, Maryland; 7 Department of International Health, Johns Hopkins Bloomberg School of Public Health, Baltimore, Maryland; 8 Department of Gynecology and Obstetrics, Johns Hopkins School of Medicine, Baltimore, Maryland; 9 Department of Pediatrics, Johns Hopkins School of Medicine, Baltimore, Maryland

## Abstract

**Importance:**

Preeclampsia is an independent risk factor for future cardiovascular disease and disproportionally affects non-Hispanic Black women. The association of maternal nativity and duration of US residence with preeclampsia and other cardiovascular risk factors is well described among non-Hispanic Black women but not among women of other racial and ethnic groups.

**Objective:**

To examine differences in cardiovascular risk factors and preeclampsia prevalence by race and ethnicity, nativity, and duration of US residence among Hispanic, non-Hispanic Black, and non-Hispanic White women.

**Design, Setting, and Participants:**

This cross-sectional analysis of the Boston Birth Cohort included a racially diverse cohort of women who had singleton deliveries at the Boston Medical Center from October 1, 1998, to February 15, 2016. Participants self-identified as Hispanic, non-Hispanic Black, or non-Hispanic White. Data were analyzed from March 1 to March 31, 2021.

**Exposures:**

Maternal nativity and duration of US residence (<10 vs ≥10 years) were self-reported.

**Main Outcome and Measures:**

Diagnosis of preeclampsia, the outcome of interest, was retrieved from maternal medical records.

**Results:**

A total of 6096 women (2400 Hispanic, 2699 non-Hispanic Black, and 997 non-Hispanic White) with a mean (SD) age of 27.5 (6.3) years were included in the study sample. Compared with Hispanic and non-Hispanic White women, non-Hispanic Black women had the highest prevalence of chronic hypertension (204 of 2699 [7.5%] vs 65 of 2400 [2.7%] and 28 of 997 [2.8%], respectively), obesity (658 of 2699 [24.4%] vs 380 of 2400 [15.8%] and 152 of 997 [15.2%], respectively), and preeclampsia (297 of 2699 [11.0%] vs 212 of 2400 [8.8%] and 71 of 997 [7.1%], respectively). Compared with their counterparts born outside the US, US-born women in all 3 racial and ethnic groups had a significantly higher prevalence of obesity (Hispanic women, 132 of 556 [23.7%] vs 248 of 1844 [13.4%]; non-Hispanic Black women, 444 of 1607 [27.6%] vs 214 of 1092 [19.6%]; non-Hispanic White women, 132 of 776 [17.0%] vs 20 of 221 [9.0%]), smoking (Hispanic women, 98 of 556 [17.6%] vs 30 of 1844 [1.6%]; non-Hispanic Black women, 330 of 1607 [20.5%] vs 53 of 1092 [4.9%]; non-Hispanic White women, 382 of 776 [49.2%] vs 42 of 221 [19.0%]), and severe stress (Hispanic women, 76 of 556 [13.7%] vs 85 of 1844 [4.6%]; non-Hispanic Black women, 231 of 1607 [14.4%] vs 120 of 1092 [11.0%]; non-Hispanic White women, 164 of 776 [21.1%] vs 26 of 221 [11.8%]). After adjusting for sociodemographic and cardiovascular risk factors, birth status outside the US (adjusted odds ratio [aOR], 0.74 [95% CI, 0.55-1.00]) and shorter duration of US residence (aOR, 0.62 [95% CI, 0.41-0.93]) were associated with lower odds of preeclampsia among non-Hispanic Black women. However, among Hispanic and non-Hispanic White women, maternal nativity (aOR for Hispanic women, 1.07 [95% CI, 0.72-1.60]; aOR for non-Hispanic White women, 0.98 [95% CI, 0.49-1.96]) and duration of US residence (aOR for Hispanic women <10 years, 1.04 [95% CI, 0.67-1.59]; aOR for non-Hispanic White women <10 years, 1.20 [95% CI, 0.48-3.02]) were not associated with preeclampsia.

**Conclusions and Relevance:**

Nativity-related disparities in preeclampsia persisted among non-Hispanic Black women but not among Hispanic and non-Hispanic White women after adjusting for sociodemographic and cardiovascular risk factors. Further research is needed to explore the interplay of factors contributing to nativity-related disparities in preeclampsia, particularly among non-Hispanic Black women.

## Key Points

**Question:**

Are maternal nativity and duration of US residence independently associated with preeclampsia among Hispanic, non-Hispanic Black, and non-Hispanic White women?

**Findings:**

In this cross-sectional study including 6096 women, US-born Hispanic, non-Hispanic Black, and non-Hispanic White women had worse cardiovascular risk profiles than their counterparts born outside the US. In addition, maternal nativity and duration of US residence were associated with preeclampsia among non-Hispanic Black women but not among Hispanic or non-Hispanic White women after adjustment for sociodemographic and cardiovascular risk factors.

**Meaning:**

These findings suggest that nativity-related disparities in preeclampsia among non-Hispanic Black women are not fully explained by nativity differences in sociodemographic and cardiovascular risk factors.

## Teaser

This cross-sectional analysis of data from the Boston Birth Cohort examines differences in cardiovascular risk factors, sociodemographic measures, and association of preeclampsia with maternal place of birth and duration of US residence among Hispanic, non-Hispanic Black, and non-Hispanic White women.

## Introduction

Hypertensive disorders of pregnancy such as preeclampsia are among the leading causes of maternal mortality in the US.^1,2^ In addition to their short-term effects, such as maternal end-organ failure, hypertensive disorders of pregnancy, particularly preeclampsia, are independent risk factors for future cardiovascular disease.^3^ In addition to these health burdens, the management of preeclampsia presents a substantial economic burden related mostly to the costs associated with prematurity, making it of both medical and public health importance.^4^

Among the factors associated with preeclampsia are race and ethnicity and maternal nativity, with non-Hispanic Black women having a higher risk than non-Hispanic White women.^5^ Evidence on the risk of preeclampsia among Hispanic women relative to non-Hispanic White women is conflicting.^6,7^ Although some studies have found Hispanic ethnicity to be independently associated with increased risk of preeclampsia,^6^ others have found Hispanic women to have a less or similar risk of preeclampsia compared with non-Hispanic White women despite poorer socioeconomic profiles.^7,8^

Previous studies^9,10^ demonstrated the association of maternal nativity and duration of US residence with preeclampsia among non-Hispanic Black women. Non-Hispanic Black women who are born outside the US tend to have a lower prevalence of preeclampsia and other cardiovascular risk factors than their US-born counterparts.^9,11^ Although these associations are well documented among non-Hispanic Black women, it is unclear whether maternal nativity and duration of US residence are independently associated with preeclampsia among women of other racial and ethnic groups. We build on these prior studies to examine the differences in cardiovascular risk factors, sociodemographic measures, and the cross-sectional association of preeclampsia with maternal nativity and duration of US residence among Hispanic, non-Hispanic Black, and non-Hispanic White women.

## Methods

### Data Source and Study Design

We used Boston Birth Cohort (BBC) data from October 1, 1998, to February 15, 2016, in this cross-sectional study. Data and analytic methods will be available on request to the Johns Hopkins Center on Early Life Origins of Disease.^12^ The BBC consists of mother-infant pairs recruited from the Boston University Medical Center, Boston, Massachusetts, and was originally designed as a case-control study to assess the genetic and environmental factors associated with preterm births.^13,14^ Therefore, the BBC oversampled mothers with preterm deliveries, making it a high-risk cohort. All eligible women who agreed to participate in the study provided written informed consent. A detailed description of the original design, recruitment process, and inclusion and exclusion criteria is described elsewhere.^9,15^ The BBC was approved by the institutional review boards of the Johns Hopkins School of Public Health and the Boston University Medical Center and receives annual approval from both institutions. Our present study is within the scope of the institutional review board approval for the cohort. This study followed the Strengthening the Reporting of Observational Studies in Epidemiology (STROBE) reporting guideline.

Of the 6188 mothers who identified as Hispanic, non-Hispanic Black, or non-Hispanic White, we excluded 92 women who did not have data on their place of birth, resulting in an analytic sample size of 6096 (eFigure in the Supplement). Of the 3157 women born outside the US, 607 (198 Hispanic, 326 non-Hispanic Black, and 83 non-Hispanic White women) did not have data on their duration of US residence and were therefore excluded from the analysis examining the association between duration of US residence and preeclampsia.

### Measures of Participant Characteristics and Main Outcome

Maternal characteristics considered in our analyses included race and ethnicity (Hispanic, non-Hispanic Black, or non-Hispanic White), age at index pregnancy (<20, 20 to <35, or ≥35 years), educational attainment (secondary school or less, General Educational Development/high school graduate, or college education/degree), parity (0, 1, or ≥2 births), prepregnancy body mass index (BMI) calculated from self-reported prepregnancy weight in kilograms divided by self-reported height in meters squared (<25.0, 25.0-29.9, or ≥30.0), smoking in the index pregnancy (no or yes), alcohol use in the index pregnancy (no or yes), and level of perceived stress (mild, moderate, or severe). Race and ethnicity were self-reported by choosing one of the options in the standard questionnaire interview.

Diagnoses of chronic hypertension (no or yes), chronic diabetes (no or yes), gestational diabetes (no or yes), and preeclampsia disorders (preeclampsia, eclampsia, and HELLP [hemolysis, elevated liver enzymes, and low platelet count] syndrome), our outcome of interest, were based on physician diagnosis and manually abstracted from the maternal prenatal charts by trained research staff (including X.H.). Preeclampsia was defined, at the time, based on the National High Blood Pressure Education Program Working Group on High Blood Pressure in Pregnancy as systolic blood pressure of at least 140 mm Hg or diastolic blood pressure of at least 90 mm Hg; proteinuria of at least 1+ on at least 2 occasions with onset after 20 weeks of gestation; or worsening chronic hypertension (systolic blood pressure, ≥160 mm Hg; diastolic blood pressure, ≥110 mm Hg).^16^

Mothers were considered US-born if they were born in any of the 50 US states, the District of Columbia, or other US territories; mothers who were born outside these regions were considered as being born outside the US. Duration of US residence for mothers born outside the US was defined as the number of years from immigration to the US to the time of the index pregnancy, categorized as less than or at least 10 years.^9,17,18^

### Statistical Analysis

Maternal characteristics and cardiovascular risk profile (chronic hypertension, chronic diabetes, and smoking) were summarized by race and ethnicity, nativity, and duration of US residence using proportions and differences tested with χ^2^ test statistics. A missing category was included for variables with missing values. We examined the cross-sectional association of preeclampsia with maternal place of birth and duration of US residence using logistic regression models adjusting for potential confounders. Model 1 was unadjusted; model 2 was adjusted for age; model 3 was additionally adjusted for educational level, marital status, and stress level; and model 4 was additionally adjusted for chronic hypertension, chronic diabetes, gestational diabetes, parity, BMI, and smoking. Predictive margins were used to obtain the age-adjusted prevalence estimates. Non-Hispanic White women were used as a reference for comparing preeclampsia rates by race and ethnicity, whereas US-born women were used as a reference in nativity-related analyses. Two separate sensitivity analyses were performed: one using a 15-year cutoff for duration of US residence, and another restricting all our analyses to nulliparous women, because chronic risk factors may be higher in women with higher parity.

All analyses were performed from March 1 to March 31, 2021, with STATA, version 16 (StataCorp LLC). A 2-sided α < .05 was used to determine statistical significance of the results.

## Results

A total of 6096 mothers with a mean (SD) age of 27.5 (6.3) years were included in this study. Of these, 2400 self-identified as Hispanic (556 [23.2%] US-born, 1844 [76.8%] born outside the US), 2699 women self-identified as non-Hispanic Black (1607 [59.5%] US-born, 1092 [40.5%] born outside the US), and 997 women self-identified as non-Hispanic White (776 [77.8%] US-born, 221 [22.2%] born outside the US).

### Sociodemographic and Cardiovascular Risk Factors by Race and Ethnicity

Compared with Hispanic and non-Hispanic Black and women, non-Hispanic White women were most likely to be 35 years or older (151 [15.1%] vs 293 [12.2%] and 394 [14.6%], respectively), to be nulliparous (510 [51.1%] vs 987 [41.1%] and 1123 [41.6%], respectively), and to report severe stress (190 [19.1%] vs 161 [6.7%] and 351 [13.0%], respectively), smoking (424 [42.5%] vs 128 [5.3%] and 383 [14.2%], respectively), and alcohol use (158 [15.8%] vs 154 [6.4%] and 294 [10.9%], respectively) (all *P* < .001). Non-Hispanic Black women, compared with Hispanic and non-Hispanic White women, were most likely to be single (1944 [72.0%] vs 1581 [65.9%] and 585 [58.7%], respectively), to have obesity (658 [24.4%] vs 380 [15.8%] and 152 [15.2%], respectively), and to have chronic hypertension (204 [7.5%] vs 65 [2.7%] and 28 [2.8%], respectively) (all *P* < .001). Compared with non-Hispanic Black and non-Hispanic White women, Hispanic women had the highest proportion with educational attainment of less than college (1914 [79.7%] vs 1648 [61.1%] vs 540 [54.2%], respectively; *P* < .001) (eTable 1 in the Supplement).

### Sociodemographic and Cardiovascular Risk Factors by Nativity

Across all 3 racial and ethnic groups, compared with women born outside the US, US-born women were more likely to be single (Hispanic women, 436 [78.4%] vs 1145 [62.1%]; non-Hispanic Black women, 1386 [86.2%] vs 558 [51.1%]; non-Hispanic White women, 516 [66.5%] vs 69 [31.2%]), to have obesity (Hispanic women, 132 [23.7%] vs 248 [13.4%]; non-Hispanic Black women, 444 [27.6%] vs 214 [19.6%]; non-Hispanic White women, 132 [17.0%] vs 20 [9.0%]), to smoke (Hispanic women, 98 [17.6%] vs 30 [1.6%]; non-Hispanic Black women, 330 [20.5%] vs 53 [4.9%]; non-Hispanic White women, 382 [49.2%] vs 42 [19.0%]), and to report severe stress (Hispanic women, 76 [13.7%] vs 85 [4.6%]; non-Hispanic Black women, 231 [14.4%] vs 120 [11.0%]; non-Hispanic White women, 164 [21.1%] vs 26 [11.8%]) (Table 1). Among Hispanic and non-Hispanic Black women, US-born women were additionally more likely to be younger than 20 years (Hispanic women, 144 [25.9%] vs 161 [8.7%]; non-Hispanic Black women, 299 [18.6%] vs 82 [7.5%]) and to report alcohol use (Hispanic women, 98 [17.6%] vs 30 [1.6%]; non-Hispanic Black women, 213 [13.3%] vs 81 [7.4%]). Among non-Hispanic Black and non-Hispanic White women, a greater proportion of women born outside the US had a college education compared with US-born women (non-Hispanic Black women, 485 [44.4%] vs 510 [31.7%]; non-Hispanic White women, 113 [51.1%] vs 323 [41.6%]). In contrast, a greater proportion of US-born Hispanic women had a college education than their counterparts who were born outside the US (130 [23.4%] vs 316 [17.1%]) (Table 1).

**Table 1.** Comparison of Maternal Characteristics by Maternal Place of Birth and Race and Ethnicity in the Boston Birth Cohort (1998-2016)

Characteristic	No. (%) of women by racial and ethnic group^a^								
	Non-Hispanic Black (n = 2699)			Non-Hispanic White (n = 997)			Hispanic (n = 2400)		
	Born in US (n = 1607)	Born outside US (n = 1092)	*P* value	Born in US (n = 776)	Born outside US (n = 221)	*P* value	Born in US (n = 556)	Born outside US (n = 1844)	*P* value
**Maternal demographic and obstetrical characteristics**									
Maternal age, y									
<20	299 (18.6)	82 (7.5)	<.001	53 (6.8)	8 (3.6)	.20	144 (25.9)	161 (8.7)	<.001
20 to <35	1143 (71.1)	781 (71.5)		608 (78.3)	177 (80.1)		369 (66.4)	1433 (77.7)	
≥35	165 (10.3)	229 (21.0)		115 (14.8)	36 (16.3)		43 (7.7)	250 (13.5)	
Parity									
0	687 (42.7)	436 (39.9)	.07	384 (49.5)	126 (57.0)	.004	249 (44.8)	738 (40.0)	.07
1	429 (26.7)	336 (30.8)		218 (28.1)	68 (30.8)		140 (25.2)	545 (29.5)	
≥2	491 (30.5)	320 (29.3)		174 (22.4)	27 (12.2)		167 (30.0)	561 (30.4)	
Preeclampsia									
No	1411 (87.8)	991 (90.7)	.02	721 (92.9)	205 (92.8)	.94	512 (92.1)	1676 (90.9)	.38
Yes	196 (12.2)	101 (9.2)		55 (7.1)	16 (7.2)		44 (7.9)	168 (9.1)	
**Cardiovascular disease risk factors**									
Chronic hypertension									
No	1476 (91.8)	1006 (92.1)	.54	749 (96.5)	214 (96.8)	.69	538 (96.8)	1779 (96.5)	.95
Yes	125 (7.8)	79 (7.2)		23 (3.0)	5 (2.3)		14 (2.5)	51 (2.8)	
Missing	6 (0.4)	7 (0.6)		4 (0.5)	2 (0.9)		4 (0.7)	14 (0.7)	
Chronic diabetes									
No	1538 (95.7)	1051 (96.2)	.44	741 (95.5)	212 (95.9)	.15	530 (95.3)	1801 (97.7)	.009
Yes	67 (4.2)	41 (3.7)		35 (4.5)	8 (3.6)		25 (4.5)	39 (2.1)	
Missing	2 (0.1)	0		0	1 (0.5)		1 (0.2)	4 (0.2)	
Gestational diabetes									
No	1527 (95.0)	1012 (92.7)	.01	731 (94.2)	208 (94.1)	.17	519 (93.3)	1728 (93.7)	.93
Yes	78 (4.9)	80 (7.3)		45 (5.8)	12 (5.4)		36 (6.5)	112 (6.1)	
Missing	2 (0.1)	0		0	1 (0.5)		1 (0.2)	4 (0.2)	
Smoking in pregnancy									
No	1270 (79.0)	1029 (94.2)	<.001	393 (50.6)	178 (80.5)	<.001	457 (82.2)	1796 (97.4)	<.001
Yes	330 (20.5)	53 (4.9)		382 (49.2)	42 (19.0)		98 (17.6)	30 (1.6)	
Missing	7 (0.4)	10 (0.9)		1 (0.1)	1 (0.5)		1 (0.2)	18 (1.0)	
BMI									
<25.0	694 (43.2)	482 (44.1)	<.001	445 (57.3)	136 (61.5)	.003	267 (48.0)	888 (48.1)	<.001
25.0-29.9	418 (26.0)	327 (29.9)		180 (23.2)	52 (23.5)		137 (24.6)	489 (26.5)	
≥30.0	444 (27.6)	214 (19.6)		132 (17.0)	20 (9.0)		132 (23.7)	248 (13.4)	
Missing	51 (3.2)	69 (6.3)		19 (2.4)	13 (5.9)		20 (3.6)	219 (11.9)	
**Social and environmental factors**									
Alcohol use in pregnancy									
No	1337 (83.2)	948 (86.8)	<.001	625 (80.5)	177 (80.1)	.27	457 (82.2)	1796 (97.4)	<.001
Yes	213 (13.3)	81 (7.4)		126 (16.2)	32 (14.5)		98 (17.6)	30 (1.6)	
Missing	57 (3.5)	63 (5.8)		25 (3.2)	12 (5.4)		1 (0.2)	18 (1.0)	
General stress									
Mild	413 (25.7)	434 (39.7)	<.001	111 (14.3)	80 (36.2)	<.001	172 (30.9)	1011 (54.8)	<.001
Moderate	950 (59.1)	528 (48.3)		494 (63.7)	112 (50.7)		304 (54.7)	736 (39.9)	
Severe	231 (14.4)	120 (11.0)		164 (21.1)	26 (11.8)		76 (13.7)	85 (4.6)	
Missing	13 (0.8)	10 (0.9)		7 (0.9)	3 (1.34)		4 (0.7)	12 (0.7)	
Educational level									
Secondary or less	433 (26.9)	207 (19.0)	<.001	155 (20.0)	28 (12.7)	.02	255 (45.9)	1011 (54.8)	<.001
GED/high school graduate	631 (39.3)	377 (34.5)		279 (36.0)	78 (35.3)		156 (28.1)	492 (26.7)	
College education/graduate	510 (31.7)	485 (44.4)		323 (41.6)	113 (51.1)		130 (23.4)	316 (17.1)	
Missing	33 (2.1)	23 (2.1)		19 (2.4)	2 (0.9)		15 (2.7)	25 (1.3)	
Marital status									
Married	177 (11.0)	480 (43.9)	<.001	218 (28.1)	137 (62.0)	<.001	94 (16.9)	615 (33.3)	<.001
Single	1386 (86.2)	558 (51.1)		516 (66.5)	69 (31.2)		436 (78.4)	1145 (62.1)	
Divorced, separated, or widowed	26 (1.6)	32 (2.9)		30 (3.9)	9 (4.1)		18 (3.2)	54 (2.9)	
Missing	18 (1.1)	22 (2.0)		12 (1.5)	6 (2.7)		8 (1.4)	30 (1.6)	

Abbreviations: BMI, body mass index (calculated as self-reported prepregnancy weight in kilograms divided by self-reported prepregnancy height in meters squared); GED, General Educational Development.

^a^ Percentages have been rounded and may not total 100.

### Sociodemographic and Cardiovascular Risk Factors by Duration of US Residence

Across all 3 racial and ethnic groups, women born outside the US with at least 10 years of US residence had a worse cardiovascular risk profile than women born outside the US with less than 10 years of US residence. Compared with women born outside the US with less than 10 years of US residence, those with at least 10 years of residence were more likely to be obese (Hispanic women, 78 of 344 [22.7%] vs 141 of 1302 [10.8%]; non-Hispanic Black women, 53 of 227 [23.4%] vs 92 of 539 [17.1%]; non-Hispanic White women, 5 of 32 [15.6%] vs 4 of 106 [3.8%]) and to report severe stress (Hispanic women, 25 of 344 [7.3%] vs 50 of 1302 [3.8%]; non-Hispanic Black women, 27 of 227 [11.9%] vs 47 of 539 [8.7%]; non-Hispanic White women, 9 of 32 [28.1%] vs 6 of 106 [5.7%]), smoking (Hispanic women, 9 of 344 [2.6%] vs 5 of 1302 [0.4%]; non-Hispanic Black women, 7 of 227 [3.1%] vs 9 of 539 [1.7%]; non-Hispanic White women, 9 of 32 [28.1%] vs 6 of 106 [5.7%]), and alcohol use (Hispanic women, 23 of 344 [6.7%] vs 64 of 1302 [4.9%]; non-Hispanic Black women, 26 of 227 [11.5%] vs 28 of 539 [5.2%]; non-Hispanic White women, 9 of 32 [28.1%] vs 8 of 106 [7.5%]) (eTable 2 in the Supplement).

Among non-Hispanic Black and non-Hispanic White women, the prevalence of chronic and gestational diabetes did not differ significantly by the duration of US residence. However, among Hispanic women, those born outside the US with at least 10 years of US residence had a higher prevalence of chronic diabetes (16 of 344 [4.7%] vs 20 of 1302 [1.5%]; *P* = .001) and gestational diabetes (42 of 344 [12.2%] vs 60 of 1302 [4.6%]; *P* < .001) compared with those with less than 10 years of US residence (eTable 2 in the Supplement).

### Preeclampsia by Race and Ethnicity

**Figure 1.**
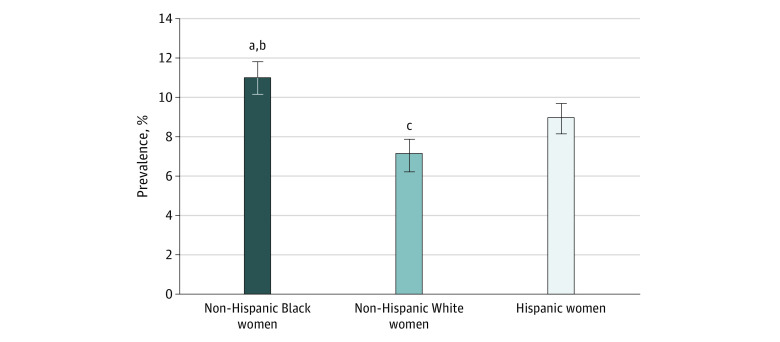
Age-Adjusted Prevalence of Preeclampsia by Race and Ethnicity Error bars indicate SEs. ^a^*P* = .001 for Non-Hispanic Black women compared with non-Hispanic White women. ^b^*P* = .01 for Hispanic women compared with Non-Hispanic Black women. ^c^*P* = .08 for Hispanic women compared with non-Hispanic White women.

The overall prevalence of preeclampsia among women included in this study was 9.5%. The age-adjusted prevalence of preeclampsia among Hispanic women was 8.9% (SE, 0.6%); among non-Hispanic Black women, 11.0% (SE, 0.6%); and among non-Hispanic White women, 7.1% (SE, 0.8%) (Figure 1). After adjusting for sociodemographic characteristics, Hispanic women (adjusted odds ratio [aOR], 1.41 [95% CI, 1.05-1.89]) and non-Hispanic Black women (aOR, 1.68 [95% CI, 1.27-2.20]) had significantly higher odds of preeclampsia compared with non-Hispanic White women. However, these differences did not persist after additionally adjusting for cardiovascular risk factors (aOR for Hispanic women, 1.16 [95% CI, 0.84-1.59]; aOR for non-Hispanic Black women, 1.17 [95% CI, 0.87-1.56]) (Table 2).

**Table 2.** Crude and Adjusted ORs for Association Between Preeclampsia and Maternal Place of Birth in the BBC (1998-2016)

Group (No. of women)	Model 1^a^		Model 2^b^		Model 3^c^		Model 4^d^	
	OR (95% CI)	*P* value	aOR (95% CI)	*P* value	aOR (95% CI)	*P* value	aOR (95% CI)	*P* value
Overall								
Non-Hispanic White (n = 997)	1 [Reference]	NA	1 [Reference]	NA	1 [Reference]	NA	1 [Reference]	NA
Non-Hispanic Black (n = 2699)	1.61 (1.23-2.11)	.001	1.62 (1.23-2.11)	.001	1.68 (1.27-2.20)	<.001	1.17 (0.87-1.56)	.30
Hispanic (n = 2400)	1.26 (0.96-1.67)	.10	1.28 (0.97-1.70)	.08	1.41 (1.05-1.89)	.02	1.16 (0.84-1.59)	.36
Non-Hispanic Black								
Born in US (n = 1607)	1 [Reference]	NA	1 [Reference]	NA	1 [Reference]	NA	1 [Reference]	NA
Born outside US (n = 1092)	0.73 (0.57-0.95)	.02	0.70 (0.54-0.90)	.006	0.74 (0.56-0.97)	.03	0.74 (0.55-1.00)	.05
Non-Hispanic White								
Born in US (n = 776)	1 [Reference]	NA	1 [Reference]	NA	1 [Reference]	NA	1 [Reference]	NA
Born outside US (n = 221)	1.02 (0.57-1.82)	.94	1.05 (0.59-1.87)	.87	0.85 (0.45-1.63)	.63	0.98 (0.49-1.96)	.95
Hispanic								
Born in US (n = 556)	1 [Reference]	NA	1 [Reference]	NA	1 [Reference]	NA	1 [Reference]	NA
Born outside US (n = 1844)	1.17 (0.82-1.65)	.38	1.11 (0.78-1.59)	.56	1.13 (0.78-1.64)	.52	1.07 (0.72-1.60)	.74

Abbreviations: aOR, adjusted odds ratio; BBC, Boston Birth Cohort; NA, not applicable; OR, odds ratio.

^a^ Unadjusted.

^b^ Adjusted for age (categorical).

^c^ Adjusted for age (categorical), educational level, marital status, and stress.

^d^ Adjusted for age (categorical), educational level, marital status, stress, chronic hypertension, chronic diabetes, gestational diabetes, parity, smoking, and body mass index.

### Preeclampsia by Nativity

**Figure 2.**
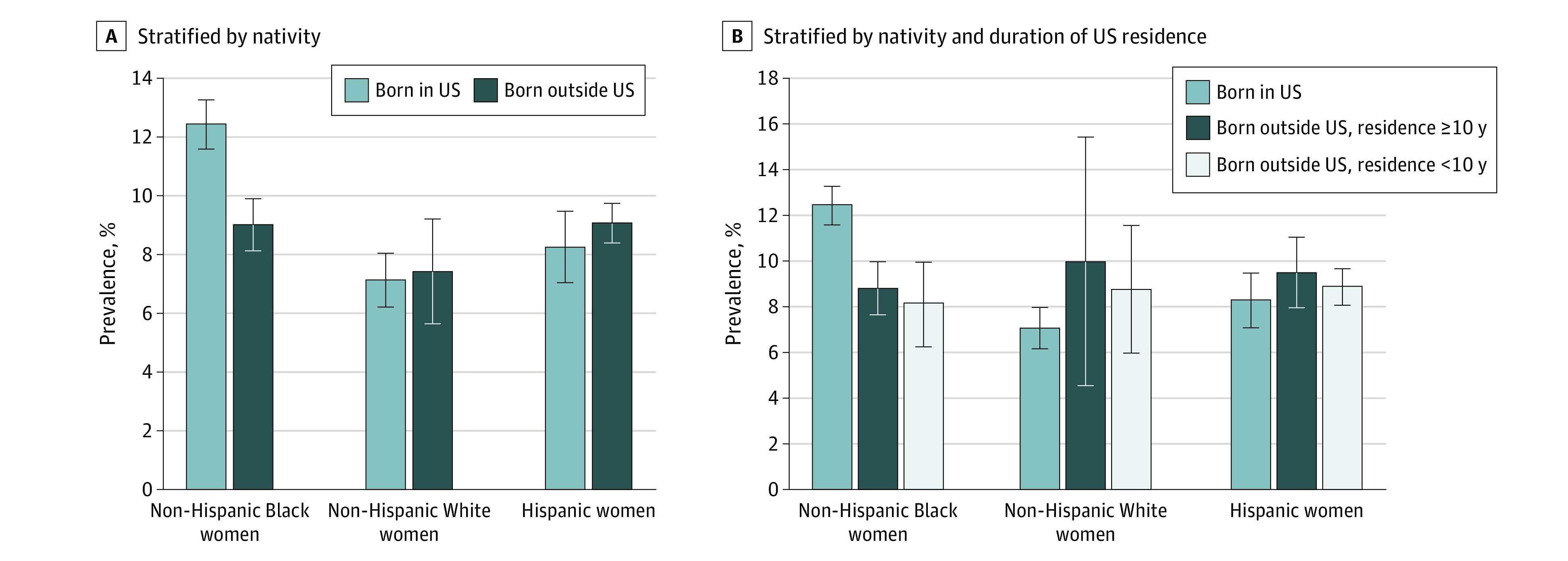
Age-Adjusted Prevalence of Preeclampsia by Nativity and Duration of US Residence Stratified by Race and Ethnicity Error bars indicate SEs.

The age-adjusted prevalence of preeclampsia among Hispanic women was 8.2% (SE, 1.2%) for those born in the US and 9.0% (SE, 0.7%) for those born outside the US (*P* = .56); among non-Hispanic Black women, 12.4% (SE, 0.8%) for those born in the US and 9.0% (SE, 0.9%) for those born outside the US (*P* = .006); and among non-Hispanic White women, 7.1% (SE, 0.9%) for those born in the US and 7.4% (SE, 1.8%) for those born outside the US (*P* = .87) (Figure 2A). After adjusting for maternal age, educational level, marital status, stress level, chronic hypertension, chronic diabetes, gestational diabetes, parity, smoking, and BMI, non-Hispanic Black women born outside the US had 26% lower odds of preeclampsia compared with US-born non-Hispanic Black women (aOR, 0.74 [95% CI, 0.55-1.00]). Hispanic women born outside the US (aOR, 1.07 [95% CI, 0.72-1.60]) and non-Hispanic White women born outside the US (aOR, 0.98 [95% CI, 0.49-1.96]) did not differ significantly in their odds of preeclampsia compared with their US-born counterparts (Table 2).

### Preeclampsia by Duration of US Residence

The age-adjusted prevalence of preeclampsia for US-born women was 8.2% (SE, 1.2%) for Hispanic women, 12.4% (SE, 0.8%) for non-Hispanic Black women, and 7.1% (SE, 0.9%) for non-Hispanic White women. For women born outside the US with at least 10 years of US residence, this prevalence was 9.4% (SE, 1.5%) for Hispanic women, 8.8% (SE, 1.9%) for non-Hispanic Black women, and 9.9% (SE, 5.4%) for non-Hispanic White women. For women born outside the US with less than 10 years of US residence, this prevalence was 8.8% (SE, 0.8%) for Hispanic women, 8.1% (SE, 1.2%) for non-Hispanic Black women, and 8.7% (SE, 2.8%) for non-Hispanic White women (Figure 2B). Although non-Hispanic Black women who were born outside the US and had been in the US for less than 10 years had 38% lower odds of preeclampsia compared with US-born non-Hispanic Black women (aOR, 0.62 [95% CI, 0.41-0.93]), non-Hispanic Black women born outside the US with at least 10 years of US residence did not differ significantly in their odds of preeclampsia compared with US-born non-Hispanic Black women (aOR, 0.64 [95% CI, 0.38-1.09]). Duration of US residence was not significantly associated with the odds of preeclampsia among Hispanic and non-Hispanic White mothers who were born outside the US (aOR for Hispanic women with residence <10 years, 1.04 [95% CI, 0.67-1.59]; aOR for non-Hispanic White women with residence <10 years, 1.20 [95% CI, 0.48-3.02]) (Table 3). Similar results were obtained using a 15-year cutoff for duration of US residence (eTable 3 in the Supplement), and restricting all analyses to nulliparous women provided similar results with the same inference (eTables 4 and 5 in the Supplement).

**Table 3.** Crude and Adjusted ORs for Association Between Preeclampsia and Duration of US Residence in the BBC (1998-2016)

Group by nativity (No. of women)	Model 1^a^		Model 2^b^		Model 3^c^		Model 4^d^	
	OR (95% CI)	*P* value	aOR (95% CI)	*P* value	aOR (95% CI)	*P* value	aOR (95% CI)	*P* value
Non-Hispanic Black								
Born in US (n = 1607)	1 [Reference]	NA	1 [Reference]	NA	1 [Reference]	NA	1 [Reference]	NA
Born outside US								
<10 y (n = 539)	0.66 (0.47-0.92)	.02	0.62 (0.44-0.88)	.007	0.66 (0.45-0.97)	.03	0.62 (0.41-0.93)	.02
≥10 y (n = 227)	0.73 (0.46-1.18)	.20	0.68 (0.42-1.10)	.12	0.69 (0.42-1.12)	.13	0.64 (0.38-1.09)	.10
Non-Hispanic White								
Born in US (n = 776)	1 [Reference]	NA	1 [Reference]	NA	1 [Reference]	NA	1 [Reference]	NA
Born outside US								
<10 y (n = 106)	1.22 (0.58-2.54)	.60	1.26 (0.60-2.64)	.54	0.94 (0.40-2.19)	.89	1.20 (0.48-3.02)	.69
≥10 y (n = 32)	1.36 (0.40-4.59)	.63	1.46 (0.43-4.96)	.55	1.31 (0.37-4.65)	.68	1.18 (0.31-4.48)	.81
Hispanic								
Born in US (n = 556)	1 [Reference]	NA	1 [Reference]	NA	1 [Reference]	NA	1 [Reference]	NA
Born outside US								
<10 y (n = 1302)	1.10 (0.76-1.58)	.62	1.08 (0.74-1.56)	.70	1.07 (0.72-1.59)	.72	1.04 (0.67-1.59)	.87
≥10 y (n = 344)	1.40 (0.89-2.22)	.15	1.16 (0.72-1.88)	.54	1.17 (0.71-1.92)	.53	1.11 (0.65-1.88)	.71

Abbreviations: aOR, adjusted odds ratio; BBC, Boston Birth Cohort; NA, not applicable; OR, odds ratio.

^a^ Unadjusted model.

^b^ Adjusted for age (categorical).

^c^ Adjusted for age (categorical), educational level, marital status, and stress.

^d^ Adjusted for age (categorical), educational level, marital status, stress, chronic hypertension, chronic diabetes, gestational diabetes, parity, smoking, and body mass index.

## Discussion

In this racially diverse cohort of low-income women, non-Hispanic Black women had the highest age-adjusted prevalence of preeclampsia compared with Hispanic and non-Hispanic White women. Furthermore, US-born women in all 3 racial and ethnic groups had worse cardiovascular risk profiles than their counterparts born outside the US. In addition, birth status outside the US and shorter duration of US residence were associated with lower odds of preeclampsia among non-Hispanic Black women but not among Hispanic and non-Hispanic White women.

Preeclampsia is a leading cause of maternal morbidity and mortality and affects approximately 1 in 25 pregnancies in the US.^19,20^ Women who develop preeclampsia have an increased risk of chronic hypertension and cardiovascular disease later in life.^21,22,23^ Results from a large meta-analysis^3^ showed preeclampsia to be independently associated with future coronary heart disease, stroke, and cardiovascular death. Offspring of preeclamptic pregnancies are also at an increased risk of high blood pressure and obesity during childhood and young adulthood.^24^ Thus, the effects of preeclampsia go beyond the immediate pregnancy period and are therefore of significant public health importance.

The prevalence of preeclampsia reported in the present study (9.5%) is higher than the reported national prevalence (3.8%-5.0%) because the BBC is a high-risk cohort that oversamples women with preterm deliveries and has a high proportion of Black women, who are disproportionately affected by preeclampsia.^20,25^ Nevertheless, the BBC is a large, racially diverse cohort with information on maternal birthplace and duration of US residence, allowing their use to study race and ethnicity– and nativity-related disparities in cardiovascular risk factors and adverse pregnancy outcomes. Racial and ethnic differences in the risk of preeclampsia have been described previously, with non-Hispanic Black women having the greatest risk.^5,25,26^ Not only are non-Hispanic Black women disproportionately affected by preeclampsia, they are also most likely to experience related complications, including long-term cardiometabolic risk.^27,28,29,30,31,32^

Non-Hispanic Black women had the highest age-adjusted prevalence of preeclampsia compared with Hispanic and non-Hispanic White women, similar to trends reported nationally.^33^ However, the racial and ethnic differences in the prevalence of preeclampsia were no longer significant after accounting for differences in sociodemographic and cardiovascular risk factors. Compared with women of other racial and ethnic groups, non-Hispanic Black women have the highest prevalence of cardiovascular risk factors, including chronic hypertension and obesity, as seen in this present study.^34,35,36^ Chronic hypertension and prepregnancy obesity are important modifiable risk factors for preeclampsia.^37^ Effective management of chronic hypertension, use of aspirin as a preventive strategy in high-risk women, and reduction in prepregnancy obesity can reduce the risk of preeclampsia among non-Hispanic Black women and potentially narrow the racial and ethnic disparity gap.

In addition, disparities in socioeconomic indexes such as income, employment, educational attainment, access to health care, and social support may partly account for the racial and ethnic disparities in cardiovascular risk and adverse pregnancy outcomes.^38,39,40^ Although not explored in this study, the stress of systemic racism, living in racially segregated neighborhoods, and experience of discrimination negatively affect the health of non-Hispanic Black women and may therefore contribute to disparities in cardiovascular risk factors and preeclampsia.^41,42,43^ Interestingly, in our study, a greater proportion of non-Hispanic White women reported moderate or severe stress than non-Hispanic Black and Hispanic women, contrary to what has been described previously.^44,45^ Most non-Hispanic Black women in our sample were single, divorced, separated, or widowed compared with Hispanic and non-Hispanic White women and therefore may not have adequate social support. Adequate social support can act as a buffer against various stressors that contribute to adverse pregnancy or health outcomes.^46^

Beyond the racial and ethnic disparities in the risk of preeclampsia, it is essential to acknowledge the heterogeneity among women of the same race and ethnicity. They may differ by various factors, including but not limited to nativity and duration of US residence. In a prior study, Boakye et al^9^ demonstrated the differential prevalence of cardiovascular risk factors and preeclampsia by nativity and duration of US residence among non-Hispanic Black women. non-Hispanic Black women born outside the US had better cardiovascular risk profiles and lower odds of preeclampsia than US-born non-Hispanic Black women. This finding has been attributed to the selection of healthy and educated women who immigrated to the US.^47^ This protective advantage of individuals born outside the US has been demonstrated among non-Hispanic Black women with shorter but not longer duration of US residence.^9^ With longer duration of US residence, non-Hispanic Black women born outside the US adopt US behaviors such as smoking, which worsen their cardiovascular risk profile and health outcomes, thus losing the protective advantage of their nativity.^48^

As has been described among non-Hispanic Black women, US-born Hispanic and non-Hispanic White women had higher prevalence of obesity, smoking, and complaints of severe stress compared with Hispanic and non-Hispanic White women born outside the US, respectively. In most racial and ethnic groups, women born outside the US tend to have lower BMI compared with their US-born counterparts.^11,48,49^ However, with a longer duration of US residence, the BMI of women born outside the US converges toward that of native-born women.^48^ Unlike what has been demonstrated among non-Hispanic Black women, Hispanic and non-Hispanic White women born outside the US did not differ in their odds of preeclampsia compared with their US-born counterparts after accounting for differences in sociodemographic and cardiovascular risk factors. In addition, duration of US residence was not associated with preeclampsia among Hispanic and non-Hispanic White women. However, among non-Hispanic Black women, nativity-related disparities persisted after accounting for differences in sociodemographic and cardiovascular risk factors. Thus, other unexplored factors such as chronic stress due to racism, health care access, and neighborhood-level factors such as segregation may drive nativity-related disparities in the risk of preeclampsia among non-Hispanic Black women.

Among non-Hispanic Black women, those born in the US tend to have a higher allosteric load (accumulation of stress over a lifetime) from prolonged exposure to systemic racism, neighborhood poverty, and residential segregation throughout their life course that negatively affects their health.^50,51^ However, non-Hispanic Black women who were born outside the US but immigrated to the US recently may be somewhat protected from deleterious effects of discrimination because they tend to settle in immigrant-concentrated residential areas with increased social support.

The findings of our study have important implications. First, although nativity-related differences in the prevalence of preeclampsia among non-Hispanic White and Hispanic women may be explained by differences in sociodemographic and cardiovascular risk factors, nativity-related disparities in preeclampsia among non-Hispanic Black women are more complicated and may be partly driven by factors such as racism and its associated stress as well as the lack of social support for native-born non-Hispanic Black women. Thus, interventions focused on stress reduction and improvements in social support may positively affect pregnancy outcomes among non-Hispanic Black women.

### Limitations

Our study has some limitations. First, because the BBC is a high-risk cohort, rates of adverse preeclampsia are comparatively higher than rates reported nationally, and our findings are not generalizable to the US population. Important social health determinants such as neighborhood characteristics, employment, and insurance that may influence race and ethnicity– and nativity-related disparities in preeclampsia were lacking in our data and hence not explored. Thus, there is the possibility of residual confounding. Also, we could not explore nativity-related disparities in preeclampsia among women of other racial and ethnic groups such as non-Hispanic Asian and Pacific Islander groups and among the various subgroups of Hispanic women (eg, Mexican, Puerto Rican, Cuban) owing to the limited sample size. Future studies are needed to explore the association between nativity and duration of US residence and preeclampsia among women of other racial and ethnic groups and within specific subgroups.

## Conclusions

The findings of this cross-sectional study suggest that factors contributing to nativity-related disparities in preeclampsia may differ by race and ethnicity. Among non-Hispanic White and Hispanic women, differences in the sociodemographic and cardiovascular risk factors explored in this study accounted for nativity differences in preeclampsia rates. However, among non-Hispanic Black women, nativity-related disparities in preeclampsia persisted after accounting for nativity differences in sociodemographic and cardiovascular risk profiles. Therefore, future studies using more granular information on important social determinants of health, such as neighborhood-level factors, are necessary to fully understand factors contributing to nativity-related disparities in preeclampsia among non-Hispanic Black women.

## Notes

Supplement. **eFigure.** Flowchart Describing Selection of the Study Sample From the Boston Birth Cohort (1998-2016) **eTable 1.** Characteristics of Study Participants Stratified by Race and Ethnicity in the Boston Birth Cohort (1998-2016) **eTable 2.** Comparison of Maternal Characteristics by Duration of US Residence and Race and Ethnicity in the Boston Birth Cohort (1998-2016) **eTable 3.** Crude and Adjusted Odds Ratios for Association Between Preeclampsia and Duration of US Residence in the Boston Birth Cohort (1998-2016) Using 15-Year Cutoff **eTable 4.** Crude and Adjusted Odds Ratios for Association Between Preeclampsia and Maternal Place of Birth Among Nulliparous Women in the Boston Birth Cohort (1998-2016) **eTable 5.** Crude and Adjusted Odds Ratios for Association Between Preeclampsia and Length of US Residence Among Nulliparous Women in the Boston Birth Cohort (1998-2016)
