# Supplementary material for: Nativity-Related Disparities in Preeclampsia and Cardiovascular Disease Risk Among a Racially Diverse Cohort of US Women
Source: JAMA Netw Open. 2021 Dec 20;4(12):e2139564. doi: 10.1001/jamanetworkopen.2021.39564 (PMC8689384; doi:10.1001/jamanetworkopen.2021.39564)
Supplement: Supplement. — eFigure. Flowchart Describing Selection of the Study Sample From the Boston Birth Cohort (1998-2016) eTable 1. Characteristics of Study Participants Stratified by Race and Ethnicity in the Boston Birth Cohort (1998-2016) eTable 2. Comparison of Maternal Characteristics by Duration of US Residence and Race and Ethnicity in the Boston Birth Cohort (1998-2016) eTable 3. Crude and Adjusted Odds Ratios for Association Between Preeclampsia and Duration of US Residence in the Boston Birth Cohort (1998-2016) Using 15-Year Cutoff eTable 4. Crude and Adjusted Odds Ratios for Association Between Preeclampsia and Maternal Place of Birth Among Nulliparous Women in the Boston Birth Cohort (1998-2016) eTable 5. Crude and Adjusted Odds Ratios for Association Between Preeclampsia and Length of US Residence Among Nulliparous Women in the Boston Birth Cohort (1998-2016) [file jamanetwopen-e2139564-s001.pdf]

Supplementary Online Content

Boakye E, Kwapong YA, Obisesan O, et al. Nativity-related disparities in preeclampsia and cardiovascular disease risk among a racially diverse cohort of US women. *JAMA Netw Open*. 2021;4(12):e2139564. doi:10.1001/jamanetworkopen.2021.39564

eFigure. Flowchart Describing the Selection of the Study Sample From the Boston Birth Cohort (1998-2016)

eTable 1. Characteristics of Study Participants Stratified by Race and Ethnicity in the Boston Birth Cohort (1998-2016)

eTable 2. Comparison of Maternal Characteristics by Duration of US Residence and Race and Ethnicity in the Boston Birth Cohort (1998-2016)

eTable 3. Crude and Adjusted Odds Ratios for Association Between Preeclampsia and Duration of US Residence in the Boston Birth Cohort (1998-2016) Using 15-Year Cutoff

eTable 4. Crude and Adjusted Odds Ratios for Association Between Preeclampsia and Maternal Place of Birth Among Nulliparous Women in the Boston Birth Cohort (1998-2016)

eTable 5. Crude and Adjusted Odds Ratios for Association Between Preeclampsia and Length of US Residence Among Nulliparous Women in the Boston Birth Cohort (1998-2016)

This supplementary material has been provided by the authors to give readers additional information about their work.

© 2021 Boakye E et al. *JAMA Network Open*.

eFigure. Flowchart Describing the Selection of the Study Sample From the Boston Birth Cohort (1998-2016)

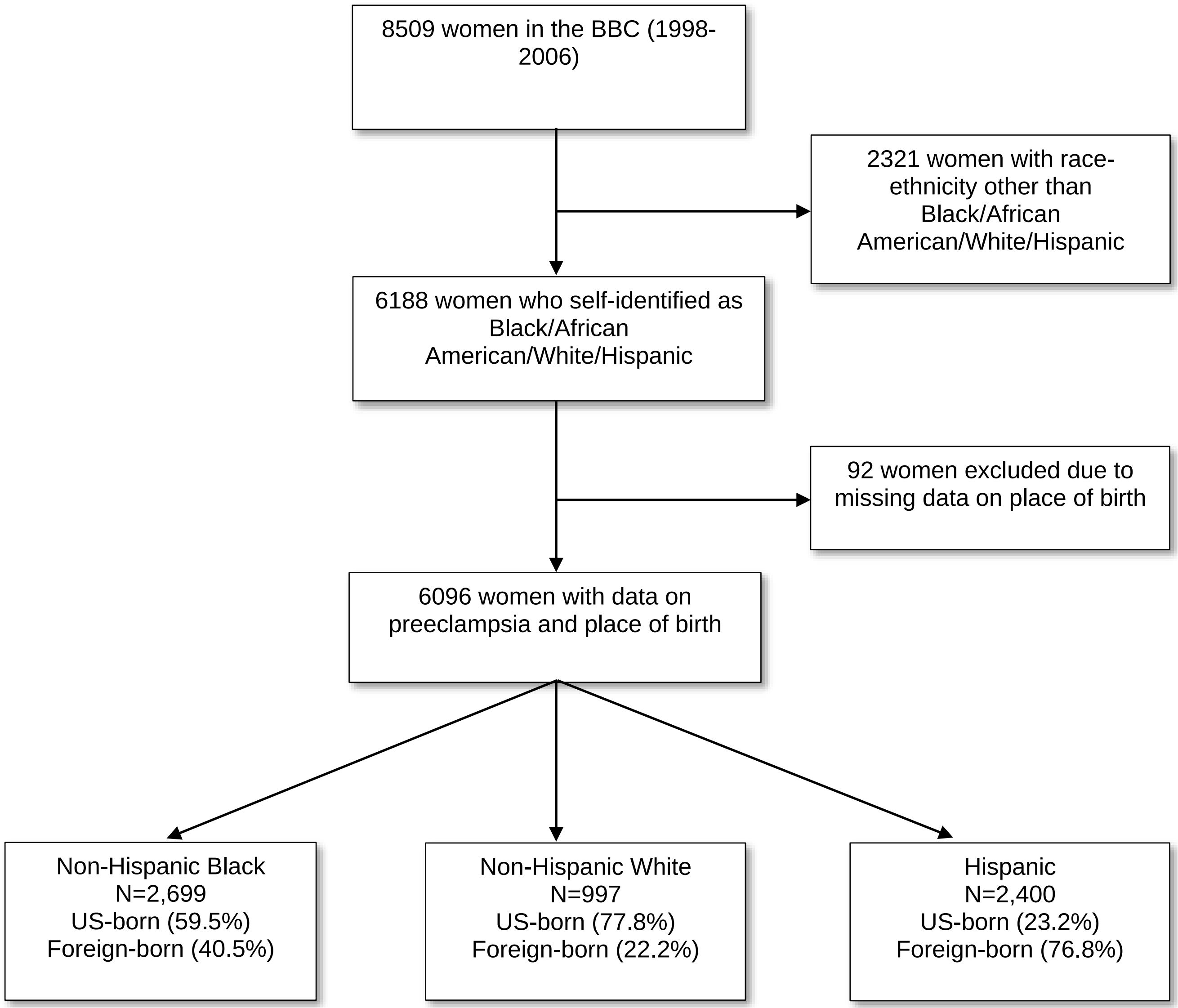

© 2021 Boakye E et al. *JAMA Network Open*.

eTable 1. Characteristics of Study Participants Stratified by Race and Ethnicity in the Boston Birth Cohort (1998-2016)

Characteristic	Total N=6096 (%)	Race-Ethnicity		
		Non-Hispanic Black N=2699 (%)	Non-Hispanic White N=997 (%)	Hispanic N=2400 (%)
Maternal Demographics and Obstetrical Characteristics				
Maternal Age, years				
<20	747 (12.3)	381 (14.1)	61 (6.1)	305 (12.7)
20-<35	4511 (74.0)	1924 (71.3)	785 (78.7)	1802 (75.1)
≥35	838 (13.7)	394 (14.6)	151 (15.1)	293 (12.2)
Parity				
0	2620 (43.0)	1123 (41.6)	510 (51.1)	987 (41.1)
1	1736 (28.5)	765 (28.3)	286 (28.7)	685 (28.6)
2+	1740 (28.5)	811 (30.1)	201 (20.2)	728 (30.3)
Preeclampsia				
No	5,516 (90.5	2402 (89.0)	926 (92.9)	2188 (91.2)
Yes	580 (9.5)	297 (11.0)	71 (7.1)	212 (8.8)
Cardiovascular Disease Risk Factors				
Chronic Hypertension				
No	5762 (94.5)	2482 (92.0)	963 (96.6)	2317 (96.5)
Yes	297 (4.9)	204 (7.5)	28 (2.8)	65 (2.7)
Missing	37 (0.6)	13 (0.5)	6 (0.6)	18 (0.8)
Chronic Diabetes				
No	5873 (96.4)	2589 (95.9)	953 (95.6)	2331 (97.1)
Yes	215 (3.5)	108 (4.0)	43 (4.3)	64 (2.7)
Missing	8 (0.1)	2 (0.1)	1 (0.1)	5 (0.2)
Gestational Diabetes				
No	5725 (93.9)	2539 (94.1)	939 (94.2)	2,247 (93.6)
Yes	363 (6.0)	158 (5.8)	57 (5.7)	148 (6.2)
Missing	8 (0.1)	2 (0.1)	1 (0.1)	5 (0.2)
Smoking in Pregnancy				
No	5123 (84.1)	2299 (85.2)	571 (57.3)	2253 (93.9)
Yes	935 (15.3)	383 (14.2)	424 (42.5)	128 (5.3)
Missing	38 (0.6)	17 (0.6)	2 (0.2)	19 (0.8)
Body Mass Index (kg/m^2)				
<25	2,912 (47.8)	1176 (43.6)	581 (58.3)	1155 (48.1)

© 2021 Boakye E et al. *JAMA Network Open*.

Characteristic	Total N=6096 (%)	Race-Ethnicity		
		Non-Hispanic Black N=2699 (%)	Non-Hispanic White N=997 (%)	Hispanic N=2400 (%)
25-29.9	1,603 (26.3)	745 (27.6)	232 (23.3)	626 (26.1)
≥30	1,190 (19.5)	658 (24.4)	152 (15.2)	380 (15.8)
Missing	391 (6.4)	120 (4.4)	32 (3.2)	239 (10.0)
Social and Environmental Factors				
Alcohol use in Pregnancy				
No	5283 (86.7)	2285 (84.7)	802 (80.4)	2196 (91.5)
Yes	606 (9.9)	294 (10.9)	158 (15.8)	154 (6.4)
Missing	207 (3.4)	120 (4.4)	37 (3.7)	50 (2.1)
General Stress				
Mild	2221 (36.4)	847 (31.4)	191 (19.1)	1183 (49.3)
Moderate	3124 (51.3)	1478 (54.8)	606 (60.8)	1040 (43.3)
Severe	702 (11.5)	351 (13.0)	190 (19.1)	161 (6.7)
Missing	49 (0.8)	23 (0.8)	10 (1.0)	16 (0.7)
Educational Level				
Secondary or less	2089 (34.3)	640 (23.7)	183 (18.4)	1266 (52.7)
GED/High School Grad	2013 (33.0)	1008 (37.3)	357 (35.8)	648 (27.0)
College education/Grad	1877 (30.8)	995 (36.9)	436 (43.7)	446 (18.6)
Missing	117 (1.9)	56 (2.1)	21 (2.1)	40 (1.7)
Marital Status				
Married	1721 (28.2)	657 (24.3)	355 (35.6)	709 (29.5)
Single	4110 (67.4)	1944 (72.0)	585 (58.7)	1581 (65.9)
Divorced/Separated/Widowed	169 (2.8)	58 (2.2)	39 (3.9)	72 (3.0)
Missing	96 (1.6)	40 (1.5)	18 (1.8)	38 (1.6)

© 2021 Boakye E et al. *JAMA Network Open*.

eTable 2. Comparison of Maternal Characteristics by Duration of US Residence and Race and Ethnicity in the Boston Birth Cohort (1998-2016)

Characteristic	Non-Hispanic Black				Non-Hispanic White				Hispanic			
	US-born N=1,607	Foreign-born ≥10 years N=227	Foreign-born <10 years N=539	p-value	US-born N=776	Foreign-born ≥10 years N=32	Foreign-born <10 years N=106	p-value	US-born N=556	Foreign-born ≥10 years N=344	Foreign-born <10 years N=1302	p-value
**Maternal Demographics and Obstetrical Characteristics**												
**Maternal Age, years**				<.001				.15				<.001
<20	299 (18.6)	21 (9.3)	18 (3.3)		53 (6.8)	0 (0.0)	3 (2.8)		144 (25.9)	18 (5.2)	117 (9.0)	
20-<35	1143 (71.1)	148 (65.2)	408 (75.7)		608 (78.4)	24 (75.0)	86 (81.1)		369 (66.4)	229 (66.6)	1051 (80.7)	
≥35	165 (10.3)	58 (25.6)	113 (21.0)		115 (14.8)	8 (25.0)	17 (16.1)		43 (7.7)	97 (28.2)	134 (10.3)	
**Parity**				.006				.01				<.001
0	687 (42.8)	79 (34.8)	234 (43.4)		384 (49.5)	14 (43.7)	66 (62.3)		249 (44.8)	84 (24.4)	580 (44.6)	
1	429 (26.7)	69 (30.4)	172 (31.9)		218 (28.1)	11 (34.4)	31 (29.3)		140 (25.2)	96 (27.9)	388 (29.8)	
2+	491 (30.5)	79 (34.8)	133 (24.7)		174 (22.4)	7 (21.9)	9 (8.5)		167 (30.0)	164 (47.7)	334 (25.6)	
**Preeclampsia**				.03				.79				.32
No	1411 (87.8)	206 (90.8)	494 (91.7)		721 (92.9)	29 (90.6)	97 (91.5)		512 (92.1)	307 (89.2)	1190 (91.4)	
Yes	196 (12.2)	21 (9.2)	45 (8.3)		55 (7.1)	3 (9.4)	9 (8.5)		44 (7.9)	37 (10.8)	112 (8.6)	
Cardiovascular Disease Risk Factors												
**Chronic Hypertension**				.71				.54				.24
No	1476 (91.8)	205 (90.3)	500 (92.8)		749 (96.5)	30 (93.8)	104 (98.1)		538 (96.8)	326 (94.8)	1265 (97.1)	
Yes	125 (7.8)	22 (9.7)	38 (7.1)		23 (3.0)	2 (6.2)	2 (1.9)		14 (2.5)	15 (4.3)	31 (2.4)	
Missing	6 (0.4)	0 (0.0)	1 (0.2)		4 (0.5)	0 (0.0)	0 (0.0)		4 (0.7)	3 (0.9)	6 (0.5)	
**Chronic Diabetes**				.88				.47				.001

© 2021 Boakye E et al. *JAMA Network Open*.

No	1538 (96.7)	219 (96.5)	516 (95.7)		741 (95.5)	32 (100.0)	101 (95.3)		530 (95.3)	328 (95.4)	1280 (98.3)	
Yes	67 (4.2)	8 (3.5)	23 (4.3)		35 (4.5)	0 (0.0)	5 (4.7)		25 (4.5)	16 (4.7)	20 (1.5)	
Missing	2 (0.1)	0 (0.0)	0 (0.0)		0 (0.0)	0 (0.0)	0 (0.0)		1 (0.2)	0 (0.0)	2 (0.2)	
**Gestational Diabetes**				.06				.99				<.001
No	1527 (95.0)	213 (93.8)	495 (91.8)		731 (94.2)	30 (93.8)	100 (94.3)		519 (93.4)	302 (87.8)	1240 (95.2)	
Gestational	78 (4.9)	14 (6.2)	44 (8.2)		45 (5.8)	2 (6.3)	6 (5.7)		36 (6.5)	42 (12.2)	60 (4.6)	
Missing	2 (0.1)	0 (0.0)	0 (0.0)		0 (0.0)	0 (0.0)	0 (0.0)		1 (0.2)	0 (0.0)	2 (0.2)	
**Smoking in Pregnancy**				<.001				<.001				<.001
No	1270 (79.0)	220 (96.9)	525 (97.4)		393 (50.6)	23 (71.9)	99 (93.4)		457 (82.2)	331 (96.2)	1286 (98.8)	
Yes	330 (20.5)	7 (3.1)	9 (1.7)		382 (49.2)	9 (28.1)	6 (5.7)		98 (17.6)	9 (2.6)	5 (0.4)	
Missing	7 (0.5)	0 (0.0)	5 (0.9)		1 (0.1)	0 (0.0)	1 (0.9)		1 (0.2)	4 (1.2)	11 (0.8)	
**Body Mass Index**				<.001				<.001				<.001
<25	694 (43.2)	103 (45.4)	234 (43.4)		445 (57.4)	14 (43.8)	73 (68.9)		267 (48.0)	135 (39.2)	672 (51.6)	
25-29.9	418 (26.0)	64 (28.2)	170 (31.5)		180 (23.2)	13 (40.6)	19 (17.9)		137 (24.6)	103 (29.9)	332 (25.5)	
≥30	444 (27.6)	53 (23.4)	92 (17.1)		132 (17.0)	5 (15.6)	4 (3.8)		132 (23.8)	78 (22.7)	141 (10.8)	
Missing	51 (3.2)	7 (3.1)	43 (8.0)		19 (2.4)	0 (0.0)	10 (9.4)		20 (3.6)	28 (8.2)	157 (12.1)	
**Social and Environmental Factors**												
**Alcohol use in Pregnancy**				<.001				.03				<.001
No	1337 (83.2)	195 (85.9)	493 (91.5)		625 (80.6)	23 (71.9)	96 (90.6)		457 (82.2)	316 (91.9)	1214 (93.2)	
Yes	213 (13.3)	26 (11.5)	28 (5.2)		126 (16.2)	9 (28.1)	8 (7.5)		98 (17.6)	23 (6.7)	64 (4.9)	
Missing	57 (3.6)	6 (2.6)	18 (3.3)		25 (3.2)	0 (0.0)	2 (1.9)		1 (0.2)	5 (1.4)	24 (1.9)	
**General Stress**												

© 2021 Boakye E et al. *JAMA Network Open*.

Mild	413 (25.7)	66 (29.1)	272 (50.5)	<.001	111 (14.3)	6 (18.8)	49 (46.2)	<.001	172 (30.9)	169 (49.1)	757 (58.1)	<0.001
Moderate	950 (59.1)	129 (56.8)	217 (40.3)		494 (63.7)	17 (53.1)	49 (46.2)		304 (54.7)	149 (43.3)	485 (37.3)	
Severe	231 (14.4)	27 (11.9)	47 (8.7)		164 (21.1)	9 (28.1)	6 (5.7)		76 (13.7)	25 (7.3)	50 (3.8)	
Missing	13 (0.8)	5 (2.2)	3 (0.5)		7 (0.9)	0 (0.0)	2 (1.9)		4 (0.7)	1 (0.3)	10 (0.8)	
**Educational Level**				<.001				.001				<.001
Secondary or less	433 (26.9)	34 (15.0)	100 (18.6)		155 (20.0)	2 (6.3)	7 (6.6)		255 (45.8)	166 (48.3)	738 (56.7)	
GED/High School Grad	631 (39.3)	83 (36.6)	187 (34.7)		279 (36.0)	10 (31.2)	35 (33.0)		156 (28.1)	94 (27.3)	351 (27.0)	
College education/Grad	510 (31.7)	110 (48.5)	250 (46.4)		323 (41.6)	20 (62.5)	63 (59.4)		130 (23.4)	81 (23.5)	207 (15.9)	
Missing	33 (2.1)	0 (0.0)	2 (0.4)		19 (2.4)	0 (0.0)	1 (1.0)		15 (2.7)	3 (0.9)	6 (0.4)	
**Marital Status**				<.001				<.001				<.001
Married	177 (11.0)	92 (40.5)	291 (54.0)		218 (28.1)	21 (65.6)	83 (78.3)		94 (16.9)	139 (40.4)	423 (32.5)	
Single	1386 (86.3)	126 (55.5)	227 (42.1)		516 (66.5)	6 (18.8)	20 (18.9)		436 (78.4)	183 (53.2)	828 (63.6)	
Divorced/Separated/Widowed	26 (1.6)	6 (2.7)	16 (3.0)		30 (3.9)	5 (15.6)	3 (2.8)		18 (3.2)	18 (5.2)	30 (2.3)	
Missing	18 (1.1)	3 (1.3)	5 (0.9)		12 (1.5)	0 (0.0)	0 (0.0)		8 (1.5)	4 (1.2)	21 (1.6)	

© 2021 Boakye E et al. *JAMA Network Open*.

eTable 3. Crude and Adjusted Odds Ratios for Association Between Preeclampsia and Duration of US Residence in the Boston Birth Cohort (1998-2016) Using 15-Year Cutoff

	**Odds Ratio (95% Confidence Interval)**							
	**Model 1**	**p-value**	**Model 2**	**p-value**	**Model 3**	**p-value**	**Model 4**	**p-value**
Non-Hispanic Black								
US-born (n=1607)	Ref		Ref		Ref		Ref	
Foreign-born <15 years (n=661)	0.61 (0.45-0.85)	.003	0.58 (0.42-0.81)	.001	0.61 (0.43-0.87)	.007	0.56 (0.38-0.82)	.003
Foreign-born ≥15 years (n=105)	1.11 (0.62-1.98)	.73	1.02 (0.57-1.84)	.94	0.98 (0.54-1.79)	.95	1.02 (0.53-1.96)	.95
Non-Hispanic White								
US-born (n=776)	Ref		Ref		Ref		Ref	
Foreign-born <15 years (121)	1.18 (0.58-2.38)	.64	1.23 (0.61-2.50)	.56	0.93 (0.41-2.09)	.86	1.12 (0.47-2.66)	.80
Foreign-born ≥15 years (n=17)	1.75 (0.39-7.84)	.47	1.85 (0.41-8.32)	.42	1.75 (0.37-8.24)	.48	1.70 (0.32-8.96)	.53
Hispanic								
US-born (n=556)	Ref		Ref		Ref		Ref	
Foreign-born <15 years (n=1531)	1.12 (0.78-1.59)	.54	1.07 (0.75-1.55)	.70	1.07 (0.73-1.57)	.74	1.03 (0.68-1.57)	.89
Foreign-born ≥15 years (n=115)	1.75 (0.94-3.26)	.08	1.37 (0.71-2.62)	.34	1.42 (0.73-2.74)	.30	1.30 (0.64-2.62)	.47
Model 1: Unadjusted<br>Model 2: Age-adjusted (categorical)								

© 2021 Boakye E et al. *JAMA Network Open*.

Model 3: Adjusted for age (categorical), education, marital status, and stress<br>Model 4: Adjusted for age (categorical), education, marital status, stress, chronic hypertension, chronic diabetes, gestational diabetes, parity, smoking, and BMI

© 2021 Boakye E et al. *JAMA Network Open*.

eTable 4. Crude and Adjusted Odds Ratios for Association Between Preeclampsia and Maternal Place of Birth Among Nulliparous Women in the Boston Birth Cohort (1998-2016)

	Odds Ratio (95% Confidence Interval)							
	**Model 1**	**p-value**	**Model 2**	**p-value**	**Model 3**	**p-value**	**Model 4**	**p-value**
Overall								
Non-Hispanic White (n=510)	Ref		Ref		Ref		Ref	
Non-Hispanic Black (n=1123)	1.62 (1.14-2.32)	.008	1.70 (1.18-2.44)	.004	1.73 (1.20-2.50)	.004	1.18 (0.79-1.75)	.41
Hispanic (n=987)	1.21 (0.83-1.76)	.32	1.27 (0.87-1.85)	.22	1.23 (0.83-1.83)	.30	0.99 (0.65-1.52)	.97
Non-Hispanic Black								
US-born (n=687)	Ref		Ref		Ref		Ref	
Foreign-born (n=436)	0.62 (0.43-0.91)	.013	0.58 (0.39-0.85)	.005	0.53 (0.35-0.82)	.004	0.59 (0.38-0.92)	.019
Non-Hispanic White								
US-born (n=384)	Ref		Ref		Ref		Ref	
Foreign-born (n=126)	1.36 (0.68-2.69)	.38	1.40 (0.70-2.78)	.34	1.21 (0.56-2.64)	.63	1.21 (0.51-2.85)	.66
Hispanic								
US-born (n=249)	Ref		Ref		Ref		Ref	
Foreign-born (n=738)	1.28 (0.77-2.13)	.33	1.21 (0.71-2.05)	.48	1.13 (0.67-1.96)	.65	1.23 (0.69-2.19)	.47
Model 1: Unadjusted								

© 2021 Boakye E et al. *JAMA Network Open*.

Model 2: Age-adjusted (categorical)<br>Model 3: Adjusted for age (categorical), education, marital status, and stress<br>Model 4: Adjusted for age (categorical), education, marital status, stress, chronic hypertension, chronic diabetes, gestational diabetes, parity, smoking, and BMI

© 2021 Boakye E et al. *JAMA Network Open*.

eTable 5. Crude and Adjusted Odds Ratios for Association Between Preeclampsia and Length of US Residence Among Nulliparous Women in the Boston Birth Cohort (1998-2016)

	**Odds Ratio (95% Confidence Interval)**							
	**Model 1**	**p-value**	**Model 2**	**p-value**	**Model 3**	**p-value**	**Model 4**	**p-value**
Non-Hispanic Black								
US-born (n=687)	Ref		Ref		Ref		Ref	
Foreign-born <10 years (n=234)	0.59 (0.36-0.96)	.03	0.54 (0.32-0.89)	.02	0.50 (0.29-0.87)	.01	0.51 (0.29-0.92)	.03
Foreign-born ≥10 years (n=79)	0.55 (0.25-1.23)	.15	0.52 (0.23-1.17)	.12	0.53 (0.23-1.24)	.14	0.61 (0.25-1.47)	.27
Non-Hispanic White								
US-born (n=384)	Ref		Ref		Ref		Ref	
Foreign-born <10 years (66)	1.86 (0.84-4.13)	.13	1.95 (0.87-4.35)	.10	1.77 (0.68-4.60)	.24	2.16 (0.71-6.56)	.17
Foreign-born ≥10 years (n=14)	1.97 (0.42-9.20)	.39	2.10 (0.44-9.92)	.35	1.85 (0.37-9.33)	.46	1.93 (0.32-11.60)	.47
Hispanic								
US-born (n=249)	Ref		Ref		Ref		Ref	
Foreign-born <10 years (n=580)	1.18 (0.70-2.00)	.53	1.10 (0.64-1.91)	.72	1.00 (0.56-1.77)	.99	1.15 (0.62-2.14)	.65
Foreign-born ≥10 years (n=84)	1.47 (0.66-3.26)	.35	1.30 (0.57-2.96)	.53	1.32 (0.58-3.05)	.51	1.35 (057-3.19)	.49
Model 1: Unadjusted								

© 2021 Boakye E et al. *JAMA Network Open*.

Model 2: Age-adjusted (categorical)
Model 3: Adjusted for age (categorical), education, marital status, and stress
Model 4: Adjusted for age (categorical), education, marital status, stress, chronic hypertension, chronic diabetes, gestational diabetes, parity, smoking, and BMI

© 2021 Boakye E et al. *JAMA Network Open*.
